# Education and Communication on the Topic of Osteonecrosis of the Jaw When Taking Bone‐Stabilizing Drugs

**DOI:** 10.1002/cre2.70024

**Published:** 2024-11-04

**Authors:** Franz Gustav Saur, Christian Keinki, Alin Cramer, Jens Buentzel, Jutta Hübner

**Affiliations:** ^1^ Medizinische Klinik II, Hämatologie und Internistische Onkologie Universitätsklinikum Jena Jena Germany; ^2^ Deutsche Krebsgesellschaft Berlin Germany; ^3^ Südharz klinikum, Klinik für HNO‐Erkrankungen Kopf‐Hals‐Chirurgie und Interdisziplinäre Palliativstation Nordhausen Germany

**Keywords:** antiresorptive agents, bisphosphonates, communication, education, osteonecrosis of the jaw, quality of life

## Abstract

**Objectives:**

The aim of this study was to analyze the communication between doctors and patients who were taking bone‐stabilizing medication and in rare cases developed osteonecrosis of the jaw as a result.

**Material and Methods:**

A standardized questionnaire recorded deficits based on patient experiences. These data were used to develop solution strategies for improving doctor–patient communication and the benefit–risk assessment of medication use.

**Results:**

Most patients were satisfied with the information provided by their doctor; however, one in three (29.8%) were not informed about possible side effects, and a quarter (24.6%) only found out about osteonecrosis of the jaw through their own research. Only half (45.7%) were asked about risk factors, and most information materials were rated poorly. The diagnosis took an average of 18.7 months, with many (47.8%) consulting a doctor only when they experienced pain. Quality of life was severely impaired, with daily pain, physical limitations, and negative effects on mental health. About a third (35.3%) reported that their quality of life had deteriorated significantly.

**Conclusions:**

Further research into patient education is necessary. Web‐based information brochures, improved follow‐up care, and close cooperation with dentists are required. The use of a running sheet, such as the AGSMO running sheet, for individual risk assessment of osteonecrosis of the jaw is recommended. Patients undergoing treatment with bone‐stabilizing medication should be monitored closely. Education about osteonecrosis of the jaw must be continued, and the medical profession must be confronted with it.

## Clinical significance

Our study contributes to presenting the disease from the patient's perspective and combines the knowledge and communication skills of doctors with patient assessments. At the same time, the study is an up‐to‐date indicator of the medical profession's knowledge of the ARONJ disease and the side effects of bone‐stabilizing medication.

## 1 Introduction

The clinical picture of osteonecrosis of the jaw is a fairly recent phenomenon (Wood et al. 2002). In 2003, it was first described that bisphosphonate‐containing drugs used in cancer therapy can cause necrosis of the jaw (Dunphy et al. 2020). As a nonhealing exposure of the jawbone, the complication was initially grouped under the term bisphosphonate‐associated osteonecrosis of the jaw (BRONJ; Reid and Cundy 2009; Ruggiero et al. 2022; Turner et al. 2016). Subsequently, it was found that not only bisphosphonates used in cancer therapy caused osteonecrosis of the jaw. Other drugs of antiangiogenic and antiresorptive drug classes administered without the addition of bisphosphonates may also cause it (Beth‐Tasdogan et al. 2022; Khan et al. 2015). These drugs can be grouped under the term bone‐modifying agents (BMAs; Eisen et al. 2022). For this reason, the broader term of antiresorptive‐related osteonecrosis of the jaw (ARONJ) was introduced (Fusco et al. 2016; Shibahara 2019).

Since 2014, the American Association of Oral and Maxillofacial Surgeons (AAOMS) has classified the severity of ARONJ into four stages. The stages 0–3 are distinguished (Aguirre, Castillo, and Kimmel 2021). In Stage 0, patients present with nonspecific symptoms and/or radiographic findings without developing clinical signs of necrotic bone. In Stage 1, probable necrotic bone is present. However, patients are asymptomatic and no signs of infection are present. Patients in Stage 2 complain of pain and signs of infection. Stage 3 adds at least one of the following features: necrotic bone spreading to adjacent regions (such as maxillary sinus, os zygomaticum, mandible), pathological fractures in the maxillary region, extraoral fistulas, mouth‐antrum junction, or extensive osteolysis (Aguirre, Castillo, and Kimmel 2021).

The pathophysiology is still not fully understood, but intensive research is constantly adding knowledge. In particular, scientific progress has been made in understanding and treatment of ARONJ in recent years. However, questions remain about the pathogenesis of the disease (Aguirre, Castillo, and Kimmel 2021). Current knowledge suggests that it is likely a multifactorial disease in which inflammation, infection, and trauma to the bone or surrounding soft tissues, induced by altered bone remodeling or excessive suppression of bone resorption combined with inhibition of angiogenesis, lead to osteonecrosis of the jaw (Lorenzo‐Pouso et al. 2021).

A consensus therapeutic strategy for the treatment of ARONJ has still not been established (Favia et al. 2018). Optimally, oncologists, dentists, and oral surgeons work closely together to evaluate each patient individually and initiate appropriate therapy (Rosella et al. 2016). In most cases, interdisciplinary exchange in daily routine proves to be difficult. Dentists in particular need to be even more closely involved in treatment (Coropciuc et al. 2017). They monitor patients' oral hygiene and are often the first to diagnose an occurring case of ARONJ.

Periodontal surgery and dentoalveolar procedures pose the main risk of maxillary necrosis (Aguirre, Castillo, and Kimmel 2021; Kuroshima et al. 2022). For prevention, it is essential to optimally adjust the timing of dental treatment and examine the teeth for a possible focus of infection. Teeth with local infection (e.g., deep probing pockets, periapical lesion, and periodontal disease) are at increased risk of ARONJ (Everts‐Graber et al. 2022; Park et al. 2023). In contrast, extraction of such teeth even while taking BMA reduces the risk (Soutome et al. 2021).

The vast majority of dentists are familiar with the term osteonecrosis of the jaw (Al‐Eid et al. 2020). The indication and mechanism of action of bisphosphonates are known to the majority. However, risk factors that cause ARONJ are unknown to the majority, similar to the concept of a “drug holiday,” in which one pauses medication to undergo dental procedures (Patil et al. 2020).

The significance of ARONJ for patients is complex. As a potentially serious condition, it is associated with many functional limitations in the oral cavity, including not only masticatory function but also swallowing and speech (Miksad et al. 2011). Therapeutic rehabilitation is challenging (Kagami et al. 2018; Ristow et al. 2019; Schiegnitz Eik et al. 2018). Patients' quality of life also decreases, whereas the level of suffering increases physically and psychologically (de Cassia Tornier et al. 2021; Murphy and Mannion 2020; Tenore et al. 2020). Detailed pre‐therapeutic education is essential and should be reasonably communicated. Regarding physician–patient communication, quality of education, and the importance of the disease, there are limited studies and data that specifically focus on these areas. Therefore, to shed light on the implications of prescribing bone‐stabilizing medications, we decided to design a questionnaire that would reveal deficiencies in upfront communication and education, as well as the physical and psychological consequences of patients, thereby assessing the impact on their quality of life. The focus is on patients' assessments and perceptions. The results can help protect patients from undesirable or even unknown risks of osteonecrosis of the jaw by providing evidence‐based and target group‐oriented information.

## 2 Methods

### 2.1 Design

The study aimed to identify how patients have been informed about possible side effects of bone‐stabilizing drugs during their treatment with a focus on the osteonecrosis of the jaw. Those affected were also contacted directly and were inquired about their quality of life.

### 2.2 Inclusion/Exclusion of Participants

The questionnaire was designed as an online questionnaire in two languages and published on January 24, 2022. The distribution of the questionnaire in German and English versions was done via the Bundesverband Deutsche Prostatahilfe e.V. and different groups of the social network Facebook. These groups were as follows: Living with Osteonecrosis of Jaw, Osteoporose Selbsthilfegruppe, Osteoporose schon Mitte 40, and Vitamin‐D Selbsthilfegruppe. The questionnaire could be accessed and completed digitally via the online portal “soscisurvey” (https://www.soscisurvey.de/). The participants had 5 weeks to answer the questionnaire. The survey period ended on March 1, 2022.

### 2.3 Questionnaire

Some questions in the questionnaire were based on the “Quality of Life Questionnaire‐Communication 26” (QLQ‐COMU26) from the European Organization for Research and Treatment of Cancer (EORTC) about patient communication and were adapted and inserted into our questionnaire (Arraras et al. 2017). The QLQ‐COMU26 questionnaire is attached to this article. The following questions from this questionnaire have been adapted and inserted for ours: No. 2, No. 6, No. 11, No. 12, No. 21, No. 24, and No. 26. Additional questions on the same topic were developed by us. The questions on quality of life and pathogenesis were also developed independently.

Closed questions were mostly used for the questionnaire. The participant could choose between different answer options. For some questions, there was the possibility to write a free answer in text form. In the sections of communication, intake period, and quality of life, participants were asked to indicate their agreement in a four‐point Likert‐type scale format (e.g., My doctor explained the reasons for my treatment: “Very true,” “Rather true,” “Rather not true,” “Not true at all”). The questionnaire is divided into two sections, which are further subdivided.

### 2.4 Medical History and Communication

1. Demographic data: (Two questions: gender, age)
2. Data on medications: (Two questions: name of medication, duration of use)
3. Data on necrosis of the jaw: (Two questions: knowledge of side effects, knowledge of necrosis of the jaw)
4. Data on informed consent: (One table: informed consent)
5. Data on intake period: (One table: communication during intake) Pathogenesis
6. Data on the course of the disease: (Two questions: onset of problem, time of diagnosis)
7. Data on severity: (One table: personal severity)
8. Data on diagnosis: (Four questions: diagnosing physician, post‐diagnosing physician, course of diagnosis, complaints)
9. Data on specialist procedure: (Three questions: doctor's response to complaints, dentist's procedure, maxillofacial procedure)
10. Data on quality of life: (One table: impact on daily life).

### 2.5 Statistics

For all data analytics, the program IBM SPSS 28 was used. Associations were carried out using *t* tests and *χ* ^2^ tests; *p* < 0.05 was considered significant.

## 3 Results

### 3.1 Demographic Data

Overall, 190 participants responded to the questionnaire, of whom 13 (6.8%) were English speakers and 177 (93.2%) were German speakers.

Of the 190 participants, 33 (17.4%) were male and 128 (67.4%) were female.

The mean age was 70 with a range from 31 to 81. Nine participants (4.6%) reported being under 50 years of age (Table 1).

**Table 1:** Demographic data (*N* = 190).

Demographic data	Number of respondents	%
Language	190	
German	177	93.2
English	13	6.8
Gender	190	
Male	33	17.4
Female	128	67.4
Divers	0	0
No answer	29	15.3
Age	190	
Under the age of 50	9	4.6
50–60	51	26.8
61–70	60	31.6
71–81	30	15.8
No answer	40	21.1

### 3.2 BMA

The majority of all participants received bisphosphonates (*n* = 77, 55.4%).

Zoledronate is the most prescribed drug in the bisphosphonate group, with 31.7% of respondents taking it (*n* = 44 of 139 participants). Denosumab was given to 52 of the 139 respondents (37.4%).

**Figure 1:**
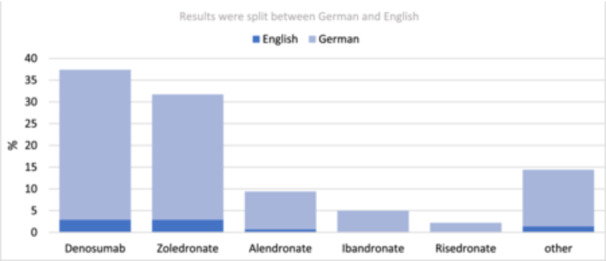
Type of bone‐modifying agent (*N* = 139).

Twenty respondents (14.4%) were taking other medications, mostly other bisphosphonates, Denosumab, and bisphosphonates in joint combination or osteoporosis drugs such as Raloxifene or Teriparatide (Figure 1).

The duration of use ranged from 1 month to over 10 years, with an average of 20 months.

Six of 154 participants (3.2%) stated that the treatment is planned, but not started yet. Twenty‐six of 154 participants (16.9%) had already discontinued their medication at the time of the survey.

Most participants received their medication intravenously (*n* = 63, 33.2%), whereas approximately one in 10 (*n* = 18, 9.5%) took it orally.

The mean treatment period after which medication was discontinued was 32.3 months, with values ranging from 1 to 96 months (*n* = 25).

### 3.3 Information About ONJ

**Figure 2:**
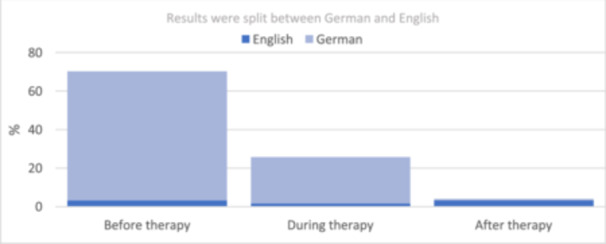
Time of information on side effects (*N* = 124).

The majority of participants were informed about the potential side effects of bone‐stabilizing medications before starting therapy (Figure 2). Eighty‐seven of 124 respondents (70.2%) reported being informed about side effects pre‐therapeutically. Thirty‐two of 124 respondents (25.8%) learned about the side effects only during treatment (Figure 2). Thus, 40% of the English‐speaking respondents only became aware of the possible side effects after discontinuing the drug. This compares with 0.8% of the German‐speaking respondents (*p* < 0.001).

When asked how patients learned about osteonecrosis of the jaw, 70 of the 122 respondents (57.4%) said they learned about the condition from their treating physician. Fourteen respondents (11.5%) were told by their dentist. Another 10 out of 122 respondents (8.1%) learned about it through friends and acquaintances, whereas 23% (*n* = 28) of all participants learned about the condition through their own research. Approximately one‐quarter of all respondents (*n* = 30, 24.6%) were not informed by their treating physicians and dentists for a long time, so they did their own research.

### 3.4 Medical Consultation and Communication

Approximately half of the respondents (*n* = 118, 55.1%) have been informed in detail about the risks of osteonecrosis of the jaw before their treatment. More than a quarter of all respondents were not informed at all (Table 2).

**Table 2:** Information on osteonecrosis of the jaw by the physician and communication during therapy (*N* = 84–118).

Statement about information on osteonecrosis of the jaw by the physician	Number of respondents	Agreement (%)			
		Very true	Rather true	Rather not true	Not true at all
My doctor gave me detailed information about the risks of jaw necrosis before treatment	118	30.5	24.6	17.8	27.1
My doctor has given me written information material	114	14	13.2	12.3	60.5
My doctor made sure I understood the information I received	112	23.3	20.5	21.4	34.8
I was satisfied with the amount of information I received	112	22.2	18.8	15.2	43.8
My doctor explained the reasons for the treatment to me	117	43.6	43.6	7.7	5.1
My doctor referred me to the dentist before treatment	117	52.1	9.4	3.5	35
Before treatment, my doctor asked about risk factors such as smoking, diabetes, or taking cortisone medications	114	24.6	21.1	24.6	29.7
My doctor gave me the opportunity to ask all my questions	115	36.5	38.3	19.1	6.1
I trusted my doctor on his recommendation	115	56.5	32.2	8.7	2.6
My doctor avoided medical terminology and used terms I could understand	115	30.4	45.2	15.7	8.7
I was worried about getting necrosis of the jaw after the clarification	114	17.5	30.7	31.6	20.2
Statement about communication during therapy	112	—	—	—	—
My doctor gave me the opportunity to ask my questions again and again	112	35.7	33.9	20.6	9.8
I was also able to raise concerns with my doctor regarding bone‐building drugs	109	31.2	34.9	23.9	10
My doctor responded sensitively to all questions	108	31.5	36.1	22.2	10.2
My doctor took my problems and concerns seriously	107	34.6	34.6	20.5	10.3
I felt that my treating doctor and dentist worked together	105	15.2	7.6	28.6	48.6
I am satisfied with my doctor's communication overall	108	27.8	37	24.1	11.1
My doctor referred me to the dentist for checkups after the treatment was completed	89	9	12.3	19.1	59.6
My doctor has continued to be available to me since the end of the treatment in case of queries	84	25	34.5	11.9	28.6

Significantly more men felt better informed about the risks than women (79% of men, 49% of women; *p* = 0.007).

Two‐thirds of all English‐speaking respondents did not feel they had received any detailed information compared with 24% of all German‐speaking respondents (*p* = 0.019).

Three statements dealt with the topic of information material. Sixty‐nine of the 114 patients (60.5%) did not receive any information material from their physician (Table 2). Thirty‐one of 112 respondents (34.8%) did not feel that their physician made sure they understood the information they received.

To the majority of respondents, the physician explained why treatment with the bone‐stabilizing drug was necessary (*n* = 102 of 117, 87.2%; Table 2). Also, most of them (*n* = 72, 61.5%) were referred to a dentist before treatment, whereas one‐third (*n* = 41, 35%) were not.

There was a mixed response to the question of whether the physician had asked about risk factors such as smoking, diabetes, or the use of cortisone preparations before the start of therapy. Each response option was selected by a quarter of the participants (Table 2).

Responding to the question of whether the physician gave patients the opportunity to ask their own questions, 74.8% of respondents agreed (*n* = 86 of 115).

**Figure 3:**
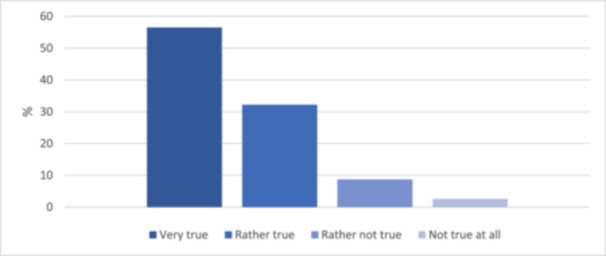
Trust in the medical profession (*N* = 115).

Trust in the medical profession is high: 65 of 115 respondents (56.5%) fully trusted their physician's recommendations, and 37 (32.2%) rated “trust in the medical profession” as rather true (Table 2 and Figure 3). All English‐speaking respondents fully trusted their doctor in this regard (*p* = 0.013).

Three‐quarters of all respondents stated that they understood the language used by the physician. When asked whether the doctor avoided medical terminology and used terms that were understandable, 75.6% of respondents (*n* = 87 of 115) fully or mostly agreed (Table 2).

Forty‐three of 114 respondents (38.2%) became afraid of developing osteonecrosis of the jaw after receiving education.

There was also an opportunity for most respondents (*n* = 78 of 112, 69.9%) to ask questions during treatment (Table 2). Similarly, there was a high level of agreement (*n* = 72 of 109, 66.1%) about the extent of concerns regarding bone‐building drugs could be expressed.

Seventy‐three of 108 participants (67.6%) felt that their doctor was empathetic (*n* = 73 of 108, 67.6%) and took them seriously (*n* = 74 of 107, 69.9%).

Finally, about six in 10 respondents (*n* = 50 of 84, 59.6%) indicated that their physician was also available to answer queries after treatment was completed, compared with 40.4% (*n* = 34) who were not (Table 2).

### 3.5 Satisfaction

**Figure 4:**
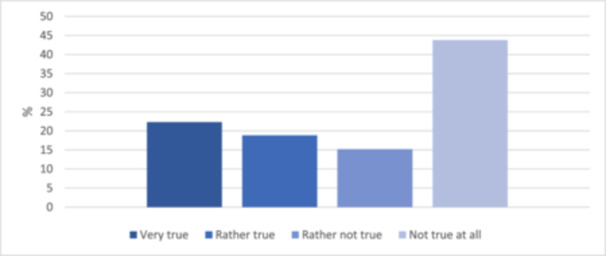
Satisfaction of received information (*N* = 112).

More than half of the respondents were dissatisfied with the scope of the information material (*n* = 112, 56.3%; Table 2 and Figure 4).

In terms of communication, 40 of 108 respondents (37%) were mostly satisfied, and 30 (27.8%) were very satisfied with their physician's communication (Table 2).

### 3.6 Osteonecrosis of the Jaw

Regarding being affected by ARONJ, most participants (*n* = 83 of 129, 64.3%) reported not being affected. Although 16.3% of respondents (*n* = 21) left the question unanswered, approximately one in five (*n* = 25) counted themselves as affected.

A significant association was found between age and ARONJ. On average, those affected by osteonecrosis of the jaw were 62.75 years old (in a range of 42–75 years; *p* = 0.035).

The onset of emerging problems varies greatly: five out of 23 respondents (21.73%) reported experiencing the first symptoms after 24 months. For the other respondents, the interval differed, sometimes greatly, and ranged from “immediately” to 10 years. On average, this amounts to 28.5 months.

The time from the onset of symptoms differed largely. The majority of respondents (*n* = 15 of 23, 65.21%) indicated a period of 0–12 months. However, in individual cases, this took up to 36 months (mean time 18.7 months).

The majority of respondents (*n* = 14 of 24, 58.3%) received their diagnosis from an oral and maxillofacial (OMF) surgeon, who subsequently was the attending physician for this side effect in most cases (*n* = 11, 52.4%). Five participants (23.8%) were treated by their dentist.

Most participants were diagnosed after reporting initial complaints (*n* = 11, 47.8%). One‐third (*n* = 8 of 23, 34.8%) asked for diagnostics of a possible ARONJ due to pain/complaints.

Eleven out of 23 respondents (47.8%) reported the complaints to their doctor immediately, whereas nearly four in 10 (*n* = 9 of 23, 39.1%) did not address them promptly.

When asked how their doctor responded to their complaints, the most common response by a fifth was that the doctor discontinued the medication or that they had been referred to the dentist (seven out of 37 [18.9%] each).

Seventy of 89 respondents, and thus the majority of respondents (78.7%), were not referred to a dentist after treatment was completed (Table 2).

Considering preventive measures, the dentist took with patients before or at the beginning of therapy, five of 37 respondents (13.5%) answered that teeth and implants not worth preserving were removed. Also, five of 37 participants (13.5%) were instructed and motivated by their dentist to practice above‐average oral hygiene.

Twelve of 37 respondents (32.4%) indicated that they were referred to an OMF surgeon by their dentist.

Upon presentation to OMF surgery, five of 37 respondents (13.5) had a bone sample taken, which was subsequently examined in the laboratory. Nearly one in five (*n* = 7, 18.9%) were advised conservative, nonsurgical treatment, whereas 10 of 37 respondents (27%) were recommended invasive surgical treatment.

### 3.7 Quality of Life

A burden, both physical and mental, was felt by most respondents to some (*n* = 6 of 15, 40%) or a high degree (*n* = 4 of 15, 26.7%; Table 3).

**Table 3:** Quality of life of those affected (*N* = 14–17).

Statement	Number of respondents	Agreement (%)			
		Very true	Rather true	Rather not true	Not true at all
I have daily pain	14	14.3	14.3	35.7	35.7
The pain affects my daily activities	14	7.1	35.7	28.6	28.6
I am impaired in eating	15	20	33.3	20	26.7
I am impaired in speaking	14	14.3	7.1	35.7	42.9
I am impaired in my facial expressions	14	21.4	7.1	28.6	42.9
Jaw necrosis has had a negative impact on my activities with family and friends	14	21.4	14.3	35.7	28.6
The necrosis of the jaw has had a negative impact on my leisure activities	15	20	20	20	40
The necrosis of the jaw has had a negative impact on my professional life	14	7.1	14.3	21.4	57.2
Jaw necrosis has had a negative impact on my mental health	15	26.7	0	46.6	26.7
Since the necrosis, I have concerns about the jaw, which often keep me busy	17	23.5	53	0	23.5
I have got into financial difficulties due to the jaw necrosis or its treatment	15	26.7	13.3	6.7	53.3
I feel a great deal of suffering because of the jaw necrosis	15	26.7	40	13.3	20
My quality of life has deteriorated considerably due to the jaw necrosis	17	35.3	17.6	29.5	17.6

A quarter of the patients (*n* = 4 of 15) stated that their mental health is negatively affected.

A significant association emerged between mental health and type of medication. Notably, 100% of all respondents taking zoledronate did not complain of any mental health problems. In contrast, 100% of all respondents taking denosumab suffered from mental health problems (*p* = 0.001).

Four of 14 respondents (28.6%) complained of daily pain, whereas six patients (42.8%) felt that they were impaired in their daily activities (Table 3).

Impairment of eating was most prominent and was stated as severely or largely impaired (*n* = 8 of 15, 53.3%). Problems with speech occurred in four out of 14 respondents (28.5%), whereas six participants (42.9%) had no problems at all.

Five out of 14 respondents (35.7%) reported that necrosis of the jaw negatively affected activities with family and friends. Six out of 15 respondents (40%) felt that their leisure time activities were disturbed and limited.

Thirteen of 17 respondents (76.5%) agreed to be preoccupied with worries about osteonecrosis of the jaw, which were complemented by financial worries for one in four (*n* = 4 of 15, 26.7%).

Finally, six of 17 respondents (35.3%) fully agreed with the statement that quality of life was significantly worsened by the necrosis of the jaw (Table 3).

No significant association was found with disease severity.

## 4 Discussion

In this study, we asked patients about their knowledge of the side effects and risks of bone stabilization therapy. Our survey showed that patients were inadequately informed about the risks and side effects of their treatment. In particular, one in three respondents reported not being informed about the potential side effects of ARONJ. These results have been documented in other studies, with the insufficient education of patients as a result of deficient medical instruction of possible risks being repeatedly denounced (Otto et al. 2018; Ruggiero et al. 2022). This also includes insufficient recording of risk factors, which were only asked of the patients in half of all cases in our study. The recording of risk factors is a simple but highly important preventive measure in the context of side effects from frequently used drugs such as ARONJ. The early identification of risk factors should lead to a faster and more comprehensive initiation of preventive measures and thus to a reduced incidence of ARONJ (Drudge‐Coates et al. 2020; Oh and Kim 2024). The doctors' communication skills revealed further deficits in the areas of conducting conversations and empathy. For one in five respondents, the language used by doctors was incomprehensible. Almost one in three were unable to communicate their concerns about bone‐stabilizing drugs and also felt unable to be taken seriously. The fact that almost 90% of respondents nevertheless trusted the recommendations of their treating doctor shows the great responsibility resting on the medical profession.

Alongside these deficits, patients were dissatisfied with the quality and scope of information material provided by doctors. This lack increased the risk of side effects arising without early supportive treatment. Moreover, patients not being fully informed are unable to make informed decisions about their treatment (Fallowfield et al. 2002). Patients try to cope with these deficits by looking for information on their own, which is underpinned in our study by the fact that one in four patients only learnt about ARONJ through their own research.

With the onset of symptoms, our respondents waited an average of 18.7 months for their diagnosis, which indicates a considerable delay in the diagnostic process. On the one hand, there is a lack of reported symptoms; on the other, patients hesitating to declare problems due to a lack of knowledge about ARONJ play a major role. Patients often consulted a doctor only after pain occurred, whereas symptoms that indicated complications were already present earlier. This problem of delay has been described by several other authors even more than a decade before our study, but no sufficient changes seem to have been made (Friedrich 2010; Gigliotti, Madathil, and Makhoul 2019). In fact, every second respondent only went to the doctor at the onset of pain. With pain being reported, a diagnosis was normally made quickly. Of respondents, 40% perceived nonpainful changes without seeing any therapeutic urgency in them. The majority of patients explained their wait‐and‐see behavior by believing the symptoms to be temporary and “not that bad,” which underlines the importance of patients' understanding and awareness of side effects. A lack of awareness of the severity and progression may lead to a delay in seeking medical help (Esther, Julius, and Deogratius 2021).

More than half of all patients were referred to an OMF surgeon, who was the one to make the final diagnosis of ARONJ. As specialists, these surgeons play a crucial role in the early detection and assessment of changes in the jaw area (Otto et al. 2018). However, dentists act as the interface between surgeons and oncologists, diagnosing one in five respondents. Collaboration with dentists plays a particularly important role, as they are often the first point of contact for patients by monitoring their oral hygiene and are crucial in the early detection of pathological changes (Otto et al. 2018). Yet, according to patient assessment, this interdisciplinary collaboration took no place in three‐quarters of all cases. This may lead to further delays in diagnosis and treatment (Ruggiero et al. 2022). In addition, 80% of all respondents did not receive a post‐therapy referral for oral hygiene checks, which is also problematic, as monitoring oral health is extremely important to detect further changes and counteract them at an early stage if necessary.

The quality of life of patients affected by is increasingly seen as a key aspect in dealing with ARONJ. This is due to the fact that patients may experience a variety of physical, emotional, social, occupational, and financial sequels (Miksad et al. 2011). Pain and difficulties in chewing are significant in the clinical picture of ARONJ and are also clearly evident in our study (Drudge‐Coates et al. 2020). Especially, chronic pain deters quality of life (Murphy and Mannion 2020; Winter et al. 2022). This in turn has an impact on the organization of everyday life activities, as our study shows. Furthermore, ARONJ imposes a significant mental burden on patients. One in four respondents indicated a negative impact on their psyche. This stress is fueled by worries that arise from ARONJ and comprise private life with partners and family, leisure activities that can no longer be carried out to the same extent as before, and may extend to professional life in some cases. In the worst case, financial worries have been reported. Consequently, the entire spectrum of an individual's life is affected, resulting in an impaired quality of life. Our observations are in line with existing research that shows that chronic pain and physical impairment may lead to depression, sleep disturbances, and family and work disagreements (Breivik et al. 2006). Such long‐term stress leads to a vicious circle in which persistent pain and uncertainty about the further course of the disease favor mental illness, which in turn has a negative impact on quality of life (Connell et al. 2012). This is underlined by the high level of suffering experienced by our respondents, with one‐third of all those affected reporting a significant deterioration in their quality of life as a result of ARONJ.

Although most doctors are aware of ARONJ, the results of our study call for important improvements to be made in patient education and for more attention to be paid to the clinical picture. As an absolute principle, the concept of “informed consent” should be followed. This means that a patient only consents to medical treatment after being fully informed of the benefits, risks, consequences, and possible alternatives. The patient must understand the information received and give their consent without coercion or pressure (Cocanour 2017). On this basis, the strategy should be to provide and use more written and digital information material that clearly and comprehensibly points out specific risks of treatment, such as ARONJ. Although web‐based information can provide additional information for younger, more tech‐savvy patient groups, it should be made available to older patients in the form of printed information leaflets or flyers. This is because written information flyers play a particularly important role for older patients, as they are less likely to use or have access to the Internet (Tesch‐Römer, Weber, and Webel 2016). In addition, older age is associated with an increased risk of ARONJ (Drudge‐Coates et al. 2020).

Another important issue is the quality of the package insert of the prescribed medication. Bauer et al. reported in their study that only one‐third of patients who read the package insert were aware of the risk of ARONJ (Bauer et al. 2012). The warnings required in the study have now been recognized as an important measure for education and prevention and are included in the package inserts for bisphosphonates and denosumab (Balkhi, Seoane‐Vazquez, and Rodriguez‐Monguio 2018). In future studies, this aspect should be queried and presented again.

The introduction of a “bisphosphonate passport” as a measure to couple psychological support with educational information, as suggested by Bauer et al. should be further considered as an addition with the inclusion of antiresorptives (Bauer et al. 2012). As an “antiresorptive passport,” detailed information about the risk of ARONJ can thus be provided in both analog and digital forms. This could also promote psychological support for patients by using the passport as continuous treatment support. This passport could also be used for adequate recording of individual risk factors, especially as the history of these factors can be taken easily and quickly (Drudge‐Coates et al. 2020). The AGSMO running sheet should be used, which is recommended in the current S3 guideline for improving interdisciplinary communication (Schiegnitz Eik et al. 2018). This provides a clear overview of the patient's individual risk of ARONJ and is immediately available to the other specialist disciplines upon referral. Moreover, in every visit, the patient should be explicitly asked whether any problems had occurred and the response should be documented.

### 4.1 Limitations

Our study has some limitations. First of all, we used different approaches to recruit participants. The questionnaire was designed in German and English and distributed, among the “Bundesverband Deutsche Prostatahilfe e.V.” and via German groups in social networks. For the English version, we chose a self‐help group in which those affected by ARONJ gathered specifically to discuss treatment concepts and advice on how to improve their condition. This range of patients corresponded exactly to the target audience of our survey and did not require further inclusion of other social group forums. In contrast, there were no German‐language self‐help groups dedicated exclusively to those affected by ARONJ. However, to reach people affected by ARONJ, we had to select self‐help groups in which it was at least possible to indirectly draw conclusions about the disease. Also, these forums were osteoporosis self‐help groups. We were able to assume that ARONJ sufferers were among their members or that there were patients who were taking or had taken bone‐stabilizing medication such as Bisphosphonates or Denosumab. It is important to note that the number of members in the self‐help groups varies greatly. Although the English‐language group had over 900 members, the German groups together had over 11,000 members. This fact also explains the large variation in the number of English‐speaking and German participants; however, this was taken into account while analyzing the data. Accordingly, there is a selection bias that may lead to those with a high level of suffering being over‐represented. It must also be taken into account that osteoporosis patients receive lower doses than patients taking antiresorptives as a result of tumor disease (Kasperk 2020). Moreover, patients who are disappointed with the communication with the physician may be over‐represented. It is conceivable that such a group could include all those affected whose education, treatment, and follow‐up by treating physician went poorly, thus favoring a severe course of ARONJ. Reasonably informed and treated patients are less visible and represented.

## 5 Conclusion

Our study makes an important contribution to presenting the disease from the patient's point of view. It combines the knowledge and communication skills of physicians with the corresponding assessments of patients. At the same time, it is a current indicator for the knowledge of the ARONJ as well as the side effects of bone‐stabilizing drugs in the medical profession.

Further research is needed, particularly in the area of patient education, to focus on satisfaction from the patient's perspective and to trace the course of development. Physician–patient communication is the basis of every therapy and should be adequately detailed and individually tailored. Knowledge of the side effects of medications is essential in this context. Increased (web‐based) information brochures and flyers are necessary for this. The high level of patient suffering also calls for improved follow‐up care and closer cooperation with dentists. The use of a running sheet listing all relevant parameters for individual risk assessment of ARONJ and leading to better interdisciplinary communication (e.g., the AGSMO running sheet) should be implemented consistently. Patients undergoing therapy with bone‐stabilizing drugs should be monitored closely. It is important that more attention is drawn to the disease of osteonecrosis of the jaw in cases of the prescription of bone‐stabilizing drugs and, thus, that medical professionals are familiar with this side effect.

## Author Contributions

**Franz Gustav Saur:** Conceptualization, project administration, investigation, data curation, formal analysis, writing–original draft. **Alin Cramer:** Writing–review & editing. **Jutta Hübner:** Conceptualization, methodology, supervision, project administration, writing–review & editing. **Christian Kinki:** Writing–review & editing. **Jens Buentzel:** Writing–review & editing.

## Ethics Statement

This study had a positive vote of the ethics committee at the University Hospital of the Friedrich Schiller University at Jena (reg. no. 2022‐2507‐Bef).

## Conflicts of Interest

The authors declare no conflicts of interest.

## Supporting information

Supporting information.

Supporting information.

## Data Availability

The data that support the findings of this study are available from the corresponding author upon reasonable request.
