# Supplementary material for: Education and Communication on the Topic of Osteonecrosis of the Jaw When Taking Bone‐Stabilizing Drugs
Source: Clin Exp Dent Res. 2024 Nov 4;10(6):e70024. doi: 10.1002/cre2.70024 (PMC11534630; doi:10.1002/cre2.70024)
Supplement: Supplementary file 1 — Supporting information. [file CRE2-10-e70024-s001.docx]

AGSMO running sheet:

Stamp of the prescriber

Date

Signature

# Referral / consultation before antiresorptive therapy of the bone with Bisphosphonates or Denosumab

## **Patient data**:.............................................................................

**Risk of necrosis of the jaw:**  •=low •=medium •=high

# Underlying disease:

 **•** primary osteoporosis

 **•** secondary/therapy-induced osteoporosis

 **•** solid tumor (mammary CA or similar) without osseous metastasis

*(Prophylaxis; Avoidance of osseous metastases)*

 • solid tumor (mammary CA, prostate CA or similar) with osseous metastasis

 • Multiple myeloma

# Special information:

Therapy urgency (e.g. pain)  high/  medium/  low Therapy Indication Tumor  curative  palliative

 **•** • antiresorptive therapy is already running, since when? ................................................

# Medication

## **Bisphosphonate:** Preparation.........................................................................

 **•** Non-amino bisphosphonate (e.g. clodronate)

 **•** Amino-bisphosphonate (e.g. zoledronate, ibandronate, alendronate). Application type:  **•** oral.  **•** i.v. interval approx. every 3 - 6 months.

 **•** i.v. interval every 12 mo.  **•** i.v. interval approx. every 4 weeks.

# Denosumab:

Application type:  **•** s.c 60 mg approx. every 6 months.

• **•** s.c 120 mg approx. every 4 weeks.

Duration of application, planned.................................................................................................................

Change of preparation:  takes place, when? .. ........... earlier preparation.. ....................................

 planned, when? ............ future preparation...................................

# further currently planned (possibly oncological) therapy

 **•** Hormone therapy, which....................................................................

 **•** Chemotherapy therapy protocol......................................................................................

…………………………………………………………………………………………….

 **•** Antibody therapy, which.................................................................................................

 **•** Immune checkpoint inhibitor therapy, which....................................................

 **•** Cortisone, duration of therapy, if applicable.......................................................................

 **•** Antiangiogenic therapy (esp. Bevacizumab), which................................

 •!! Head and neck radiotherapy *(do not enter other planning target volumes!)*

Dose............................................. Fractionation...............................................................

 Other relevant medication.......................................................................

**Dental status** (*filled in by the dentist or oral and maxillofacial surgeon*)

 **•** no dental/surgical therapy necessary

 **•** carious lesions  Therapy is done

 **•** implant  **•** Peri-implantitis  Therapy is done

 **•** mucosal-supported prostheses  **•** Pressure  Therapy is done

 new prosthetic restauration required, if necessary when? .........................  Therapy is done

 **•** Periodontitis  Therapy is done

 **•** other germ contaminants, which?...............................................................  Therapy is done

 **•** upcoming tooth extractions, if necessary which / when? ............................  Therapy is done

*other recommendations please note on the back*

Stamp Dentist/ OMF

Recommended recall intervals:  3 mo.,  6 mo.,  12months Date: Signature:

E. Schiegnitz, Mainz; K.A. Grötz, Wiesbaden; [www.agsmo.de](http://www.agsmo.de/) oder [www.onkosupport.de](http://www.onkosupport.de/)

AGSMO running sheet recommended by the S3 guideline AR-ONJ (007-091; [www.awmf.de)](http://www.awmf.de/)

Translated from german
