# Supplementary material for: Education and Communication on the Topic of Osteonecrosis of the Jaw When Taking Bone‐Stabilizing Drugs
Source: Clin Exp Dent Res. 2024 Nov 4;10(6):e70024. doi: 10.1002/cre2.70024 (PMC11534630; doi:10.1002/cre2.70024)

ENGLISH

**EORTC QLQ-COMU26**

We are interested in the communication you have had with the professional(s) who treat you.

Please indicate among the following categories of professional(s) the one that is evaluated:

Doctor(s)

Nurse(s)

Psychologist(s)

Radiotherapy technician(s)

Other (please specify: _____________)

Please think about your communication with these professionals as you answer the questions.

Please indicate among the following periods the one you are evaluating. Please choose only one

period of time.

During the period of diagnosis

treatment

follow up

Please think about your experiences during these periods as you answer the questions.

Please answer ALL the questions yourself by circling the number that best applies to you. There are

no right or wrong answers. The information that you provide will remain strictly confidential.

**Not at**

**All**

**A**

**Little**

**Quite**

**a Bit**

**Very**

**Much**

1

.

Have you had enough opportunities to talk with your

professional(s)?

1

1

1

2

2

2

3

3

3

4

4

4

2

3

4

.

.

.

Have you felt free to ask questions?

Have you had the opportunity to express your emotions?

Have you felt that you and your professional(s) had a shared

understanding of your disease and treatment?

1

1

1

1

1

2

2

2

2

2

3

3

3

3

3

4

4

4

4

4

5

6

7

8

9

.

.

.

.

.

Has there been mutual trust between you and your professional(s)?

Have your professional(s) spent enough time talking with you?

Have your professional(s) approached you as an equal?

Have your professional(s) shown sincerity?

Have your professional(s) made it easy for you to talk openly

about issues that concern you?

1

1

1

2

2

2

3

3

3

4

4

4

1

1

0. Have your professional(s) treated you with respect?

1. Have your professional(s) taken your problems seriously?

Please go on to the next page


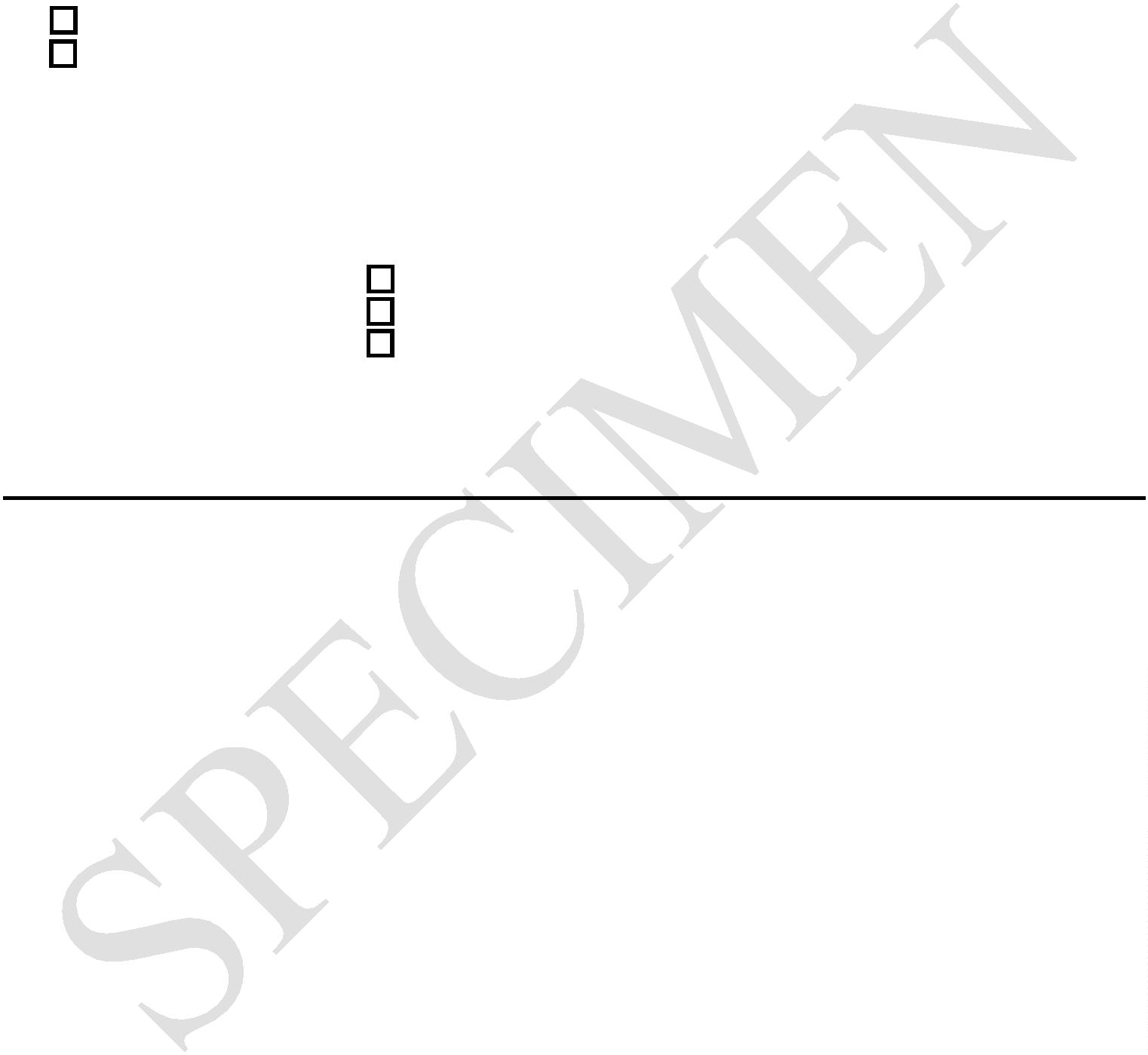

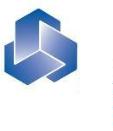

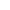

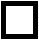

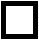

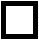


ENGLISH

**Not at**

**All**

**A**

**Little**

**Quite**

**a Bit**

**Very**

**Much**

1

2. Have your professional(s) used language that you understand

(avoided medical jargon, used clear terms)?

1

1

1

1

2

2

2

2

3

3

3

3

4

4

4

4

1

1

1

1

3. Have your professional(s) answered your questions openly?

4. Have your professional(s) looked at you during conversation?

5. Have your professional(s) used a calm voice?

6. Have your professional(s) tried to understand

your current situation?

1

1

1

1

1

1

1

2

2

2

2

2

2

2

3

3

3

3

3

3

3

4

4

4

4

4

4

4

1

1

1

2

2

2

2

7. Have your professional(s) listened to you

when you expressed your emotions?

8. Have your professional(s) helped you to manage your emotions:

for example sadness, anger, fear, anxiety and others?

9. Have your professional(s) taken into account how

you prefer to receive information*?*

0. Have your professional(s) checked what you already knew

about your illness before giving you any new information?

1. Have your professional(s) checked your understanding

of information you have been given?

2. If you did not understand the information provided,

did your professional(s) explain it again in a different way?

N/A

3. Have your professional(s) answered difficult questions

(for example concerns about your future) in a way

you felt was sufficient for you?

1

2

3

4

2

4. Have your professional(s) explained to you the aims

of the treatment (for example to control symptoms) in a way

you felt was sufficient for you?

1

1

1

2

2

2

3

3

3

4

4

4

2

2

5. Has there been enough privacy to have confidential conversations

with your professional(s)?

6. Have you been satisfied with your communication with

your professional(s)?

©

Copyright 2013 EORTC Quality of Life Group. All rights reserved. Phase IV module.


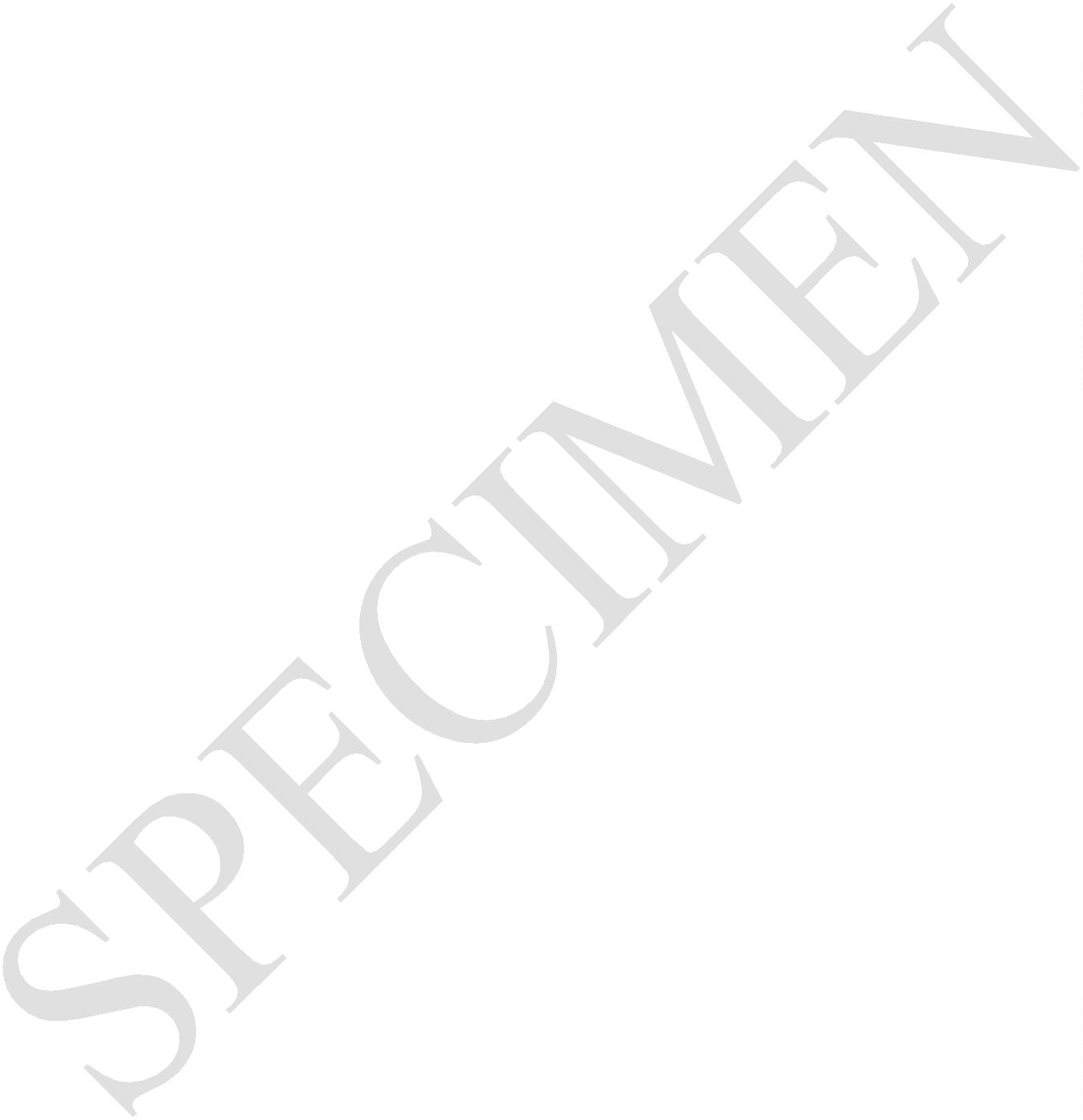

Supplement: Supplementary file 2 — Supporting information. [file CRE2-10-e70024-s002.docx]
